# The Cytokinesis-Block Micronucleus Assay on Human Isolated Fresh and Cryopreserved Peripheral Blood Mononuclear Cells

**DOI:** 10.3390/jpm10030125

**Published:** 2020-09-14

**Authors:** Simon Sioen, Karlien Cloet, Anne Vral, Ans Baeyens

**Affiliations:** Radiobiology, Department of Human Structure and Repair, Ghent University, Corneel Heymanslaan 10, 9000 Gent, Belgium; Simon.Sioen@ugent.be (S.S.); Karlien.Cloet@gmail.com (K.C.); Anne.Vral@ugent.be (A.V.)

**Keywords:** PBMCS, micronucleus assay, biological dosimetry, human blood, genotoxicity tests, radiosensitivity

## Abstract

The cytokinesis-block micronucleus (CBMN) assay is a standardized method used for genotoxicity studies. Conventional whole blood cultures (WBC) are often used for this assay, although the assay can also be performed on isolated peripheral blood mononuclear cell (PBMC) cultures. However, the standardization of a protocol for the PBMC CBMN assay has not been investigated extensively. The aim of this study was to optimize a reliable CBMN assay protocol for fresh and cryopreserved peripheral blood mononuclear cells (PBMCS), and to compare micronuclei (MNi) results between WBC and PBMC cultures. The G_0_ CBMN assay was performed on whole blood, freshly isolated, and cryopreserved PBMCS from healthy human blood samples and five radiosensitive patient samples. Cells were exposed to 220 kV X-ray in vitro doses ranging from 0.5 to 2 Gy. The optimized PBMC CBMN assay showed adequate repeatability and small inter-individual variability. MNi values were significantly higher for WBC than for fresh PBMCS. Additionally, cryopreservation of PBMCS resulted in a significant increase of MNi values, while different cryopreservation times had no significant impact. In conclusion, our standardized CBMN assay on fresh and cryopreserved PBMCS can be used for genotoxicity studies, biological dosimetry, and radiosensitivity assessment.

## 1. Introduction

Chromosomal damage can be assessed using various cytogenetic assays. These assays are often used in environmental biomonitoring studies, for example studies evaluating the genotoxic impact of nanoparticles on chromosomal damage, and in occupational genotoxicity studies, such as the influence of anesthetic gases in medical workers or the effect of cadmium exposure on battery manufacture workers [1,2,3,4]. Next to this, mutagen sensitivity phenotyping, measured by quantifying genotoxic events induced by chemical or physical agents, has been used as an indirect measure of cancer susceptibility [5,6]. Furthermore, the measurement of chromosomal damage induced by ionizing radiation is frequently used for radiation protection (e.g., biological dosimetry), as well as for radiobiological research (e.g., in vitro chromosomal radiosensitivity studies and prediction, or the follow up of radiation side effects) [3,7,8].

Ionizing radiation affects cells directly and indirectly, and can lead to an increase of the frequency of biological phenomena such as cell death, malignant transformations and chromosomal aberrations [9,10,11]. Cytogenetic biological dosimetry measures radiation-induced chromosomal aberrations as a proxy to estimate a received dose [12,13]. Over the past few decades, the use of chromosomal damage biomarkers has proven to be of value for dose assessments, especially when physical dosimetry data is insufficiently available [13].

The cytokinesis-block micronucleus (CBMN) assay, developed by Fenech and Moreley, is one of the most important in vivo and in vitro cytogenetic methods, next to the comet assay and the dicentric chromosome assay [3,14,15]. Micronuclei (MNi) are small, extra nuclear bodies, that form as a result of whole chromosomes that lag behind during mitosis or as a result of chromosome fragments that are not incorporated into the main daughter nuclei. During the CBMN assay, cytokinesis is blocked by the addition of cytochalasin B, resulting in the formation of binucleated (BN) cells. In these BN cells, which have gone through a single division, the MNi are counted [7,15,16]. The CBMN assay is faster and requires less specialized skills than most cytogenetic methods. This makes the CBMN assay a highly suitable high-throughput method for the biomonitoring of large populations exposed to genotoxic agents in both occupational and environmental settings [3,15].

The CBMN assay, performed on whole blood, is a well-established technique. Samples can be easily obtained through venipuncture, and whole blood cultures require minimal effort to set up, which leads to a reduction in time and cost [3]. One disadvantage of working with fresh whole blood samples is that in most cases only a small amount of blood can be collected. Therefore, repeating the assay is not easy without further blood sampling [17]. Furthermore, fresh blood samples need to be processed within 48 h after collection [18]. To counter these drawbacks, the CBMN assay is now more commonly performed on peripheral blood mononuclear cells (PBMCS) [19,20]. PBMCS, including monocytes and lymphocytes, can be isolated in bulk (1–2 × 10^6^ PBMCS/mL) from anticoagulated blood. One fraction can be used immediately for multiple assays while another fraction can be frozen for future tests [21,22,23,24]. While freshly isolated PBMCS samples are optimal for many downstream applications, the cryopreservation of PBMCS allows for batching and analysis within a chosen time frame [24,25]. Moreover, the use of cryopreserved PBMCS allows the analysis of sequential samples from the same patient isochronously [23].

Despite the frequent use of PBMCS, there has not been a thorough comparative analysis of CBMN assay results, between whole blood cultures, cryopreserved PBMCS, and freshly isolated PBMCS. In this study, the CBMN assay methodology for fresh and cryopreserved PBMCS was optimized and results were compared to whole blood CBMN assay results. Furthermore, the peripheral blood mononuclear cell (PBMC) CBMN assay was applied for radiosensitivity assessments and retrospective biological dosimetry.

## 2. Materials and Methods

### 2.1. Study Population and Blood Sampling

For the comparison of the whole blood and PBMC CBMN assays, blood samples from ten healthy volunteers (five men and five women; 22–53 years) were collected. To investigate the effect of cryopreservation of PBMCS on MNi counts; blood samples from three extra volunteers (two women and one man; 24–44 years) were collected. To validate the PBMC micronucleus assay for radiosensitivity assessment, blood samples from two patients with a known ataxia telangiectasia mutation (ATM), one patient with a heterozygote ATM mutation, one patient with a BRCA2 mutation and one with an unknown mutation, were collected. Repeatability of the optimized PBMC CBMN assay was verified using blood samples from 6 volunteers (3 men and 3 women; 24–43). To test the minimal blood volume for the PBMC MN assay, blood samples from 3 volunteers were collected (1 man and 2 women; 24–46). Lastly, the suitability of the PBMC CBMN assay for biological dosimetry was examined using blood samples from three extra volunteers (1 man and 2 women; 24–26 years).

All peripheral blood samples were collected by venipuncture in lithium heparinized tubes, without the use of extra stabilizers. Each sample was stored at room temperature (20 °C) and was processed within two hours of collection. Informed consent for inclusion was received from all patients and volunteers before participating in the study. The study was conducted in accordance with the Declaration of Helsinki, and the protocol was approved by the Ethics Committee of Ghent University (code: 2017/1621 and 2019/0461). None of the blood donors in this study had any known prior exposure to chemicals, radiotherapy, medication, or other substances that could affect MNi frequencies.

### 2.2. Isolation and Cryopreservation of Peripheral Blood Mononuclear Cells

#### 2.2.1. Isolation by Density Gradient Centrifugation

Peripheral blood samples were isolated using density-gradient centrifugation (2300 rpm, 20 min, 20 °C, no brakes, Eppendorf, Hamburg, Germany) of diluted blood (1/2 in phosphate buffered saline (PBS) (20 °C)), gently added on to Lymphoprep^TM^ (density: 1.077 g/mL, 20 °C, Axis-Shield, Dundee, UK). The yield and viability of the isolated PBMCS were verified using trypan blue (Gibco, Thermo Fisher Scientific Ltd., Waltham, MA, USA) and a Bürker cell counting chamber (Superior Marienfield, Lauda-Königshofen, Germany). A viability of 95–100% was attained, prior to culturing or cryopreservation. On average 1 mL blood resulted in 1.15 × 10^6^ isolated PBMCS.

#### 2.2.2. Cryopreservation and Thawing Procedures

PBMCS were cryopreserved in fetal calf serum (FCS) (4°, Gibco, Thermo Fisher Scientific Ltd., Waltham, MA, USA) supplemented with 10% dimethylsulfoxide (DMSO) (4 °C, Sigma-Aldrich, Saint Louis, MO, USA), at a concentration of 1.5 × 10^6^ cells/mL. After 24 h in a Mr. Frosty container (Sigma-Aldrich) at −80 °C, cryovials were transferred to liquid nitrogen (−196 °C).

Cells were cryopreserved for 2; 5; 10; 20 and 25 w. Thawing of cells was performed by submerging the cryovials in sterile H_2_O (37 °C) until only little pieces of ice remained. Then, the cell suspension was transferred to complete RPMI (cRPMI) [37 °C, RPMI 1640 (Gibco, Thermo Fisher Scientific Ltd., Waltham, MA, USA) with antibiotics (50 U/mL penicillin and 50 mg/mL streptomycin, Gibco)], supplemented with 10% FCS at 37 °C. This was followed by rinsing the cryovials with 1 mL of pre-warmed FCS (37 °C). Lastly, the cells were washed three times with cRPMI medium (1500 rpm, 10 min, 37 °C).

All pre-analytical factors used for PBMC-based assays are documented according to the recent standards determined by Betsou et al. [23].

### 2.3. Whole Blood Cytokinesis-Block Micronucleus Assay

**Figure 1:**
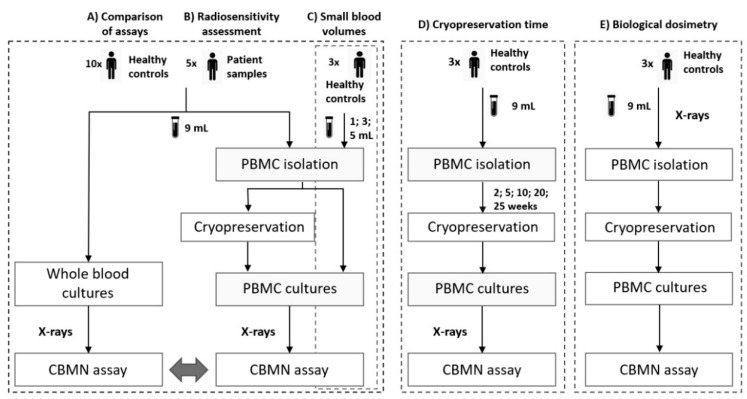
Schematic overview of experiments: comparison between the whole blood and peripheral blood mononuclear cells (PBMC) cytokinesis-block micronucleus (CBMN) assays, performed on (**A**) healthy controls and (**B**) patient samples. (**C**) Effect of different blood volumes on CBMN assay results. (**D**) The effect of different cryopreservation times on CBMN assay results. (**E**) The PBMC CBMN assay performed in a biological dosimetry setting.

The whole blood CBMN assay was performed according to a standardized protocol of our laboratory [7] (Figure 1A,B). For each culture, 0.5 mL of blood was added to 4.5 mL of pre-warmed culture medium [cRPMI + 10% FCS, 37 °C]. Following a 1-h incubation step (37 °C, 5% CO_2_), cells were irradiated with 0.5; 1 and 2 Gy X-rays in a T25 culture flask. A sham-irradiated control (0 Gy) was included to measure spontaneously occurring MNi. Duplicate cultures were set up for each dose point.

After irradiation, 20 µL/mL of phytohaemagglutanin-L (PHA-L, stock solution 1 mg/mL, Sigma-Aldrich, Saint Louis, MO, USA) was added to stimulate cell division in lymphocytes. At 23 h post-stimulation, 6 µg/mL cytochalasin B (stock solution 1.5 mg/mL, Sigma-Aldrich) was added to block cytokinesis. 70 h post-stimulation, cells were exposed to a cold hypotonic shock using 7 mL of KCl (4 °C, 0.075M, Sigma-Aldrich) and fixed using a methanol: glacial acetic acid: ringer solution (4 °C, 4:1:5, Chem-lab, Zedelgem, Belgium). After overnight storage at 4 °C, cells were fixed two more times using a methanol: glacial acetic acid solution (4:1, 4 °C). Lastly, the cells were dropped on to isopropanol cleaned slides and stained with acridine orange (10 μg/mL, Sigma-Aldrich).

### 2.4. PBMC Cytokinesis-Block Micronucleus Assay

Fresh or thawed PBMCS were resuspended in culture medium [cRPMI, 10% FCS, 1% sodiumpyruvate (100 mM, Gibco, Thermo Fisher Scientific Ltd., Waltham, MA, USA), 0.1% β-mercaptoethanol (50 mM, Gibco), 37 °C] at a concentration of 500 000 cells/mL, and were cultured in 500 µL in a 48-well cell suspension plate (Greiner Cellstar^®^, Sigma-Aldrich, Saint Louis, MO, USA) (Figure 1A–E). Following a 1-h incubation step (37 °C, 5% CO_2_), the cells were exposed to 0.5; 1 and 2 Gy X-rays. A sham-irradiated control (0 Gy) was included. For each dose point, duplicate cultures were set up.

Immediately after irradiation, 5 µL/mL of PHA-L (stock solution 1 mg/mL) was added. At 23 h post-stimulation, 6 µg/mL cytochalasin B (stock solution 1.5 mg/mL) was added. For harvesting and fixing 70 h post-stimulation, the cell suspension was transported to a 1.5 mL Eppendorf tube (Greiner Bio-one, Kremsmünster, Austria) and the well was rinsed with 0.5 mL PBS, to prevent loss of cells.

After centrifugation (8 min, 300 g, Eppendorf, Hamburg, Germany) and supernatant removal, 450 μL KCl (4 °C, 0.075M) was added to induce a cold hypotonic shock. Next, cells were fixed in a methanol: glacial acetic acid: ringer solution (3:1:4, 4 °C). After overnight storage at 4 °C, the cells were fixed two more times using a methanol: glacial acetic acid solution (3:1, 4 °C). Addition of the fixatives was always performed drop by drop while vortexing. The cells were dropped on to isopropanol cleaned slides and stained with acridine orange stain (10 μg/mL, Sigma-Aldrich, Saint Louis, MO, USA). To assure an optimal cell size for scoring MNi, fixating twice with the methanol: glacial acetic acid solution (3:1, 4 °C) is crucial.

To analyze repeatability, the PBMC CBMN assay (freshly and cryopreserved) was performed on 6 parallel cultures from three donors.

### 2.5. PBMC Cytokinesis-Block Micronucleus Assay in a Biodosimetry Setting

5 mL of whole blood was irradiated in vitro with 0; 0.5; 1 and 2 Gy X-Rays, in a T25 culture flask (Figure 1E). A sham-irradiated control (0 Gy) was included. For each dose point, duplicate cultures were set up. After a 2-h incubation step (37 °C, 5% CO_2_), PBMC isolation, cryopreservation, and thawing procedures were performed as described in Section 2.2. Subsequently, the CBMN assay was performed as described in Section 2.4, excluding the irradiation step.

### 2.6. In Vitro Irradiation Procedure

In vitro irradiations were performed at the ‘Small Animal Radiation Research Platform’ (SARRP, XStrahl, Camberley, UK) at Infinity Lab, Ghent University. X-rays (220 kVp, 13 mA), were filtered using 0.15 mm copper and a square field collimator (100 × 100 mm at 35 cm FSD) was used. The applied dose rate was 3.046 Gy/min.

### 2.7. Scoring Procedure

#### 2.7.1. Micronuclei Scoring

MNi were scored in 500 BN cells by using a fluorescence microscope (200x magnification). A total of 1000 BN cells were scored per sample as duplicated cultures were set up. Each sample was scored by two independent scorers.

#### 2.7.2. Binucleated Yield and Nuclear Division Index

To assure a sufficient proliferative capacity, the nuclear division index (NDI) was calculated. For each culture 500 viable cells (Ntotal) were scored to evaluate the number of mononucleate (N1), binucleate (N2), trinucleate (N3), and polynucleate (N4) cells. The formula used to calculate NDI: NDI= (N1 + 2N2 + 3N3 + 4N4)/Ntotal. All cultures attained NDI’s between 1.2 and 2.0.

### 2.8. Statistical Analysis

Statistical analysis was performed using the Graphpad Prism 6 software (GraphPad Software Inc., San Diego, CA, USA)and MEDCALC^®^ software (MedCalc Software Ltd., Ostend, Belgium). The confidence level of the statistical tests was 95% and statistical significance was set at *p* < 0.05. For the comparison of MNi values between different groups, the paired t test was used. Effects of cryopreservation time on MNi values were analyzed by the Friedman test. Comparison of differences in coefficients of variation (CVs) were analyzed according to Forkman [26].

## 3. Results

### 3.1. Comparison of CBMN Assay Results of Whole Blood, Fresh and Cryopreserved PBMCS

**Figure 2:**
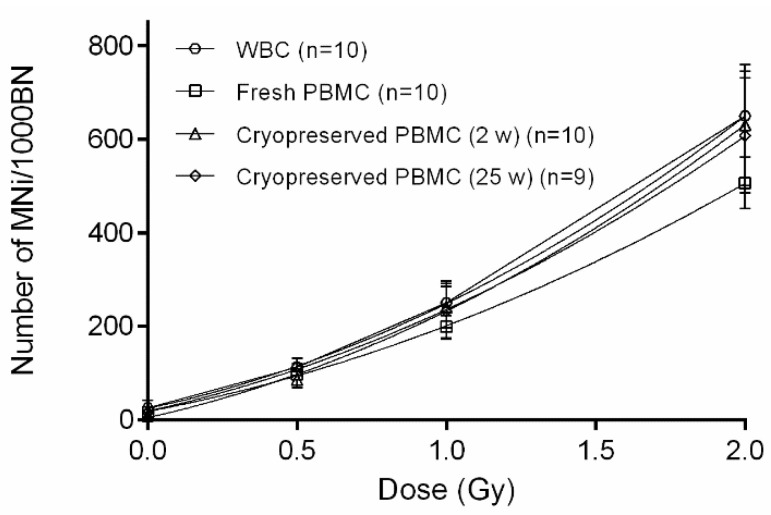
Comparison of the number of micronuclei (MNi) per 1000 binucleated (BN) cells for whole blood cultures (WBC), fresh, 2 weeks, and 25 weeks cryopreserved peripheral blood mononuclear cells (PBMCS) after 0, 0.5, 1 and 2 Gy exposure. The error bars represent SD of the mean.

The CBMN assay was performed on WBC, fresh PBMC and cryopreserved (2 w and 25 w) PBMC samples (Figure 1A). The results are presented in Table 1 and Figure 2. The mean MNi response of WBC was significantly higher compared to fresh PBMCS (paired *t*-test, *p* < 0.0001). Moreover, the mean MNi response of fresh PBMCS was significantly lower compared to both 2 w and 25 w cryopreserved PBMC samples (paired *t*-test, *p* = 0.0111 and *p* = 0.0104). When comparing both 2 w and 25 w cryopreserved PBMC samples, no significant differences were observed.

To evaluate inter-individual variability, the CV values were analyzed and compared. Although high CV-values were observed in sham-irradiated samples, due to low counted MNi values, low CV values were observed in irradiated samples. No major significant differences in CV values between the different assays were found, for each dose point (Table 1, Forkman test). However, a significant difference in inter-individual variability was noted for the 25 w cryopreserved PBMCS 1 Gy dose, compared to fresh PBMCS. In addition, the repeatability of the newly optimized PBMC CBMN assay was examined by performing the CBMN assay (2Gy) on 6 parallel cultures of three donors. The coefficient of variation for the repeats ranged between 9.8 and 11.8% for the fresh PBMC CBMN assay and between 5.7 and 10.5% for the cryopreserved PBMC CBMN assay. This indicated a good repeatability of both assays.

### 3.2. Effect of Cryopreservation Time on PBMC CBMN Assay Results

**Figure 3:**
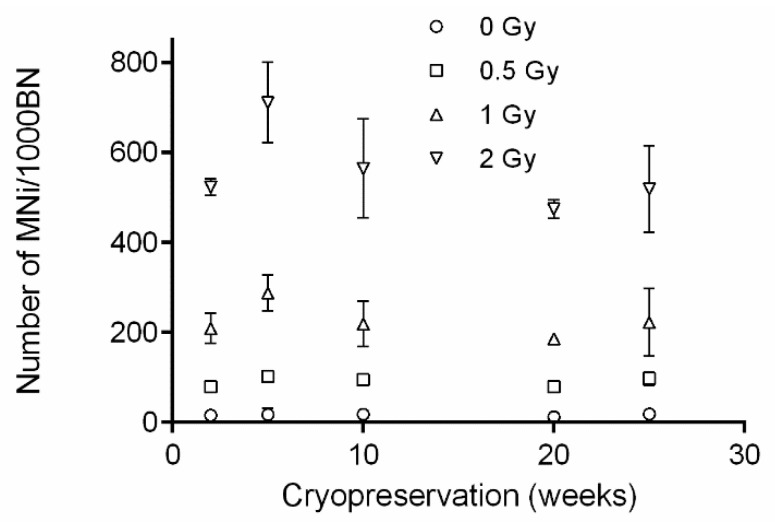
Effect of cryopreservation time (2, 5, 10, 20 and 25 weeks) on the number of micronuclei (MNi) per 1000 binucleated (BN) cells in peripheral blood mononuclear cells (PBMCS). The error bars represent SD of the mean.

The influence of cryopreservation time on MNi values was investigated as significantly higher MNi values were observed for cryopreserved PBMCS compared to fresh PBMCS. The CBMN assay was performed on cryopreserved PBMCS (2, 5, 10, 20 and 25 weeks) from three healthy donors (Figure 1D). In Figure 3, the numbers of MNi per 1000 BN cells are plotted in function of the cryopreservation time. No significant differences between 2, 5, 10, 20 and 25 w of cryopreservation were found for each dose point.

### 3.3. Radiosensitivity Assessment of Patient Samples Using the PBMC CBMN Assay

**Figure 4:**
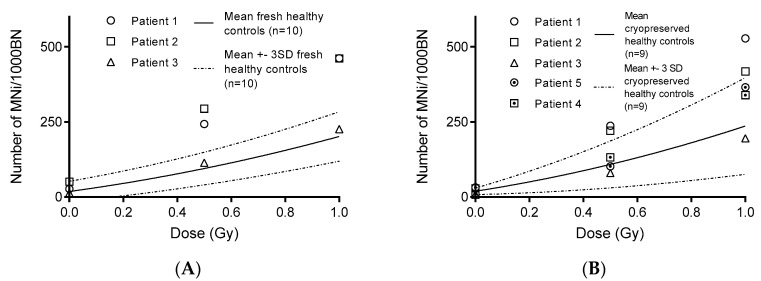
Radiosensitivity assessment of (**A**) fresh and (**B**) 25 weeks cryopreserved peripheral blood mononuclear cells (PBMCS) of 5 patients, exposed to 0, 0.5 and 1 Gy. Patients are regarded as being radiosensitive when their micronuclei (MNi) values exceed the mean +3 SD of a healthy control group, for each dose. According to clinical data, Patients 1 and 2 were considered radiosensitive while Patients 3, 4 and 5 were not radiosensitive.

To investigate whether the CBMN assay on freshly isolated and on 25 w cryopreserved PBMCS can be utilized for radiosensitivity assessment, five blood samples of patients were analyzed using the CBMN assay on WBC, freshly isolated (*n* = 3), and cryopreserved (*n* = 5; 25 w) PBMCS (Figure 1B). When evaluating WBC in a clinical setting, patients are regarded as being radiosensitive when their MNi values exceed the mean +3SD of a healthy control group, for each dose. The MNi results obtained from the freshly isolated and cryopreserved (25 w) PBMC CBMN assay were plotted against the mean ±3SD linear quadratic fits of their corresponding healthy controls (Figure 4A,B). Based on the clinical data, Patients 1 and 2 were considered radiosensitive while Patients 3, 4 and 5 were not radiosensitive. These diagnoses were confirmed by the results of our optimized PBMC CBMN assay, performed on both freshly isolated and cryopreserved (25 w) PBMCS. These results demonstrate the ability of the PBMC CBMN assay to correctly assess radiosensitivity.

### 3.4. The CBMN Assay on PBMCs, Isolated from Small Blood Volumes

**Figure 5:**
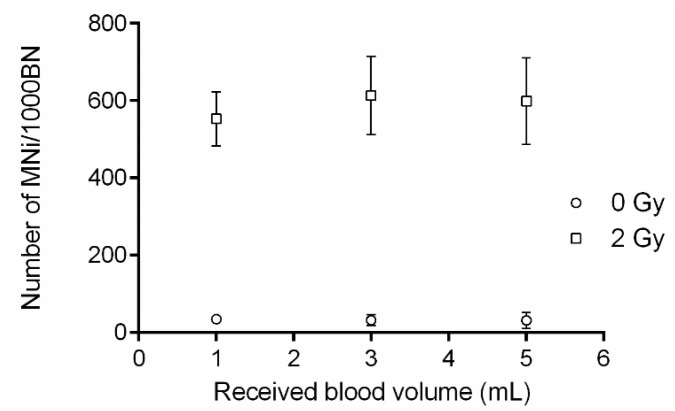
Evaluation of the volume of blood required for a peripheral blood mononuclear cell (PBMC) cytokinesis-block micronucleus (CBMN) assay. PBMC isolation was performed on 1 mL, 3 mL and 5 mL of blood and the obtained PBMCS were subsequently used for the CBMN assay.

The blood volume required for a PBMC CBMN assay was evaluated, as small blood volumes are often preferred in pediatric patients (Figure 5). PBMC isolation was performed on 1 mL, 3 mL and 5 mL of blood and the obtained PBMCS were subsequently used for the CBMN assay (Figure 1C). PBMC isolation on all blood volumes was successful. Moreover, no significant differences in MNi values were observed between the different blood volumes.

### 3.5. The CBMN Assay on PBMCS in a Biodosimetry Setting

**Figure 6:**
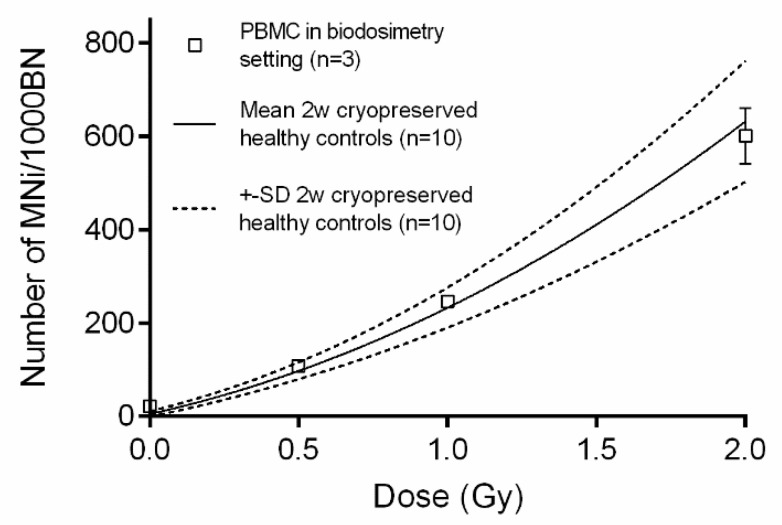
Whole blood was exposed to 0.5, 1 and 2 Gy, before peripheral blood mononuclear cell (PBMC) isolation. The micronuclei (MNi) mean ± SD of three donors is plotted against the linear quadratic fit of the mean ± SD of ten cryopreserved (2 w) PBMC controls.

In cytogenetic biological dosimetry, a received dose of ionizing radiation based on the measurement of radiation-induced biological effects, is estimated retrospectively [12,13]. In the given situation, PBMCS are irradiated in whole blood and can only be isolated afterwards. Therefore, a simulation of this scenario was performed to test the PBMC CBMN assay (Figure 1E). Results indicate that, in a biodosimetry setting, the CBMN assay is reliable to estimate the received dose (Figure 6). The MNi response of 2 w cryopreserved PBMCS fit within the mean ± SD of 10 cryopreserved (2 w) controls, for each dose point.

## 4. Discussion

While WBC are commonly used for cytogenetic assays, isolated PBMCS are also finding their way in chromosomal damage research [21,22,23]. The potential for cryopreservation of PBMCS makes this an exceptionally suitable cell type for cytogenetic assays, as it has several major advantages: (1) the ability to obtain a bulk of cells; (2) no need for further blood sampling; (3) the potential for batching and (4) the ability to perform the analysis within a chosen time frame. However, the use of cryopreserved PBMC samples for cytogenetic assays, such as the CBMN assay, has not yet been standardized and extensively studied. In this study, the CBMN assay was optimized for freshly isolated and cryopreserved PBMCS. The repeatability, inter-individual variability and sensitivity of the optimized PBMC CBMN assay was investigated. Furthermore, a thorough comparison was performed between the conventional whole blood CBMN assay and the optimized PBMC CBMN assay, on both freshly isolated and cryopreserved PBMCS.

Our results showed a significantly higher MNi responses in WBC compared to fresh PBMCS. These results may indicate the occurrence of changes in DNA damage response resulting from different culturing methods. Compared to whole blood cultures, PBMC cultures do not contain blood serum (including micronutrients), erythrocytes, platelets, or granulocytes, and they have a different cytokine composition. These differences can contribute to higher MNi values observed in whole blood [27,28,29,30]. In addition, the use of 0.1% β-mercaptoethanol in PBMC cultures might lead to a decrease in radiation-induced reactive oxygen species and consequently less DNA damage.

Importantly, as no significant differences were observed in the repeatability and inter-individual variability of both CBMN assays, the difference in MNi response has no direct impact on the reliability of the optimized PBMC CBMN assay. Interestingly, a significant increase in the MNi response of cryopreserved PBMCS was observed when exposed to irradiation. This increase, however, was independent of the cryopreservation time. Moreover, the repeatability and inter-individual variability did not vary significantly after cryopreservation, indicating no significant change in the reliability of the assay. In current literature, several studies have investigated the link between the cryopreservation of human lymphocytes and DNA damage induction and repair. Both Ho et al. and Koppen et al. reported no significant differences in DNA damage induction between fresh and cryopreserved PBMCS or whole blood samples, which is in line with our results [31,32]. Duthie et al. demonstrated that DNA damage that is already present in the DNA of fresh lymphocytes, is maintained throughout the isolation and cryopreservation procedure. Furthermore, a diminished DNA repair capacity of cryopreserved lymphocytes was reported, when exposed to H_2_O_2_ [33]. These comet assay observations are confirmed by our results, as an increased number of radiation-induced MNi was observed in our cryopreserved PBMCS. Noteworthy, the comet assay was often used in the current literature, however this technique differs from the CBMN assay. The CBMN assay analyses chromosomal fragments resulting from unrepaired or misrepaired DNA DSBs, whereas the comet assay is a straightforward visual method used for the detection of both repairable and unrepairable DNA damage [34]. Further supporting our results, Cheng et al. demonstrated that lymphocytes, from cryopreserved whole blood, were significantly more sensitive to chromatid break induction by γ irradiation, while no significant differences were seen between the baseline levels of chromatid breaks after cryopreservation [35]. In addition, and also worth mentioning, Fowke et al. showed that lymphocytes from cryopreserved whole blood, have a significantly increased probability to suffer from deterioration in terms of viability, apoptosis, and Epstein–Barr virus transformability [36].

Interestingly, Zijno et al. investigated the suitability of cryopreserved PBMCS (18 to 123 w) for the CBMN assay and found no significant differences in MNi counts between cryopreserved PBMCSs and WBC, for both the untreated and irradiated group. However, a direct comparison with our results is not straightforward as different cryopreservation protocols were used and their cryopreserved PBMCS data were compared with mean values of fresh whole blood data from five years earlier [37]. Furthermore, they observed no significant impact of different cryopreservation times, which is in line with our results. In the event that cryopreservation had led to a decrease in damage, this could have been explained by the predominant loss during storage and thawing of more unstable cells [31]. This is further supported by Risom and Knudsen, who stated that cryopreservation of lymphocytes may result in the selective survival of lymphocytes with relatively high DNA repair activity [38].

When performing a radiosensitivity assessment of a patient, it is important to keep in mind that our results indicate a possible effect of the process of cryopreservation on the occurrence of DNA damage. To counter this, we advise to simultaneously process PBMCS of a healthy control sample. Furthermore, as stated by various research groups, care should be taken regarding differences in isolation protocols and storage conditions for all PBMC samples in one study. In addition, it is important to provide a precise description of preanalytical processing and variations [23,32].

Our results indicated that radiosensitivity assessment of patients can be performed using the PBMC CBMN assay. A study of Djuzenova et al. in which PBMCS were used to demonstrate that cancer patients with an adverse skin reaction to radiotherapy displayed increased frequencies of both spontaneous and radiation-induced MN, supports our results [39]. Additional experiments using patients with different genetic defects affecting radiosensitivity, are still needed to fully confirm our hypothesis. The possibility to perform the CBMN assay on cryopreserved PBMCS provides a major advantage as the need for further blood sampling can be prevented. Moreover, we also demonstrated the potential to perform the assay on smaller blood volumes (down to 1 mL), which is especially important for pediatric patients.

Finally, this study showed the ability of the PBMC CBMN assay to be applied to retrospective biological dosimetry, in which the individual absorbed dose after a radiological accident can be estimated.

## 5. Conclusions

In conclusion, this study presents an optimized, reliable CBMN assay for both fresh and cryopreserved PBMCS. There is a significant difference between the MNi values of WBC and freshly isolated PBMCS. Furthermore, cryopreservation of PBMCS has an impact on the MNi yield. Finally, fresh or cryopreserved PBMCS provide us with a good cell model for CBMN assays in the field of biological dosimetry and radiosensitivity assessments.

## Figures and Tables

**Table 1:** Overview of the micronuclei (MNi) results of the different MN assays. Mean, SD and range values represent number of MNi/1000 binucleated (BN) cells. CVs represent the inter-individual variability.

	WBC (*n* = 10)				Fresh PBMCS (*n* = 10)			
Dose (Gy)	0	0.5	1	2	0	0.5	1	2
Mean	25	113	251	650	17	98	199	507
SD	16	18	47	95	10	23	24	55
CV (%)	63	16	19	15	57	24	12	11
Range	13–66	93–155	179–348	506–815	3–33	73–142	166–232	401–594
	**Cryopreserved (2 w) PBMCS (*n* = 10)**				**Cryopreserved (25 w) PBMCS (*n* = 9)**			
Dose (Gy)	0	0.5	1	2	0	0.5	1	2
Mean	9	86	241	630	17	114	232	608
SD	6	17	44	129	6	18	59	123
CV (%)	71	20	18	21	39	16	26 *	20
Range	2–22	67–115	190–338	494–943	11–29	82–135	158–350	424–793

* Significant *p* value (<0.05, Forkman) for the comparison of WBC to fresh PBMCS or fresh PBMCS compared to cryopreserved PBMCS.

## Abbreviations

CBMN	Cytokinesis-block micronucleus
MNi	Micronuclei
BN	Binucleated
FISH	Fluorescence in situ hybridization
PBMCS	Peripheral blood mononuclear cells
PBS	Phosphate buffered saline
FCS	Fetal calf serum
DMSO	Dimethylsulfoxide
cRPMI	Complete RPMI
PHA-L	Phytohaemagglutanin-L
SARRP	Small animal radiation research platform
NDI	Nuclear division index
CV	Coefficient of variation
WBC	Whole blood cultures
w	Weeks
DSB	Double stranded break
